# Association Between Free Triiodothyronine to Free Thyroxine Ratio and Hemorrhagic Transformation After Intravenous Thrombolysis in Acute Ischemic Stroke Patients

**DOI:** 10.1002/brb3.71140

**Published:** 2025-12-17

**Authors:** Haoran Ma, Hong Li, Xinyi Fu, Chenchen Wei, Aijun Ma

**Affiliations:** ^1^ Department of Neurology The Affiliated Hospital of Qingdao University Qingdao China

**Keywords:** acute ischemic stroke, FT3/FT4 ratio, hemorrhagic transformation, intravenous thrombolysis

## Abstract

**Background:**

Hemorrhagic transformation (HT) is a critical complication following intravenous thrombolysis (IVT) in acute ischemic stroke (AIS). Emerging evidence indicates thyroid homeostasis’ potential role in cerebrovascular outcomes, with the free triiodothyronine to free thyroxine (FT3/FT4) ratio demonstrating superior predictive value over isolated thyroid hormone measurements. This observational study works on elucidating the relation between pre‐treatment FT3/FT4 ratios and post‐thrombolytic HT occurrence in AIS populations.

**Method:**

The research consecutively enrolled AIS patients receiving IVT therapy between January 2020 and August 2024. Clinical information from patients was gathered, and to examine the link between the FT3/FT4 ratio and post‐IVT HT, we performed both multivariate and univariate logistic regression analyses. The optimal cutoff value was identified through the ROC curve analysis. The relation between clinical outcomes in HT patients and the FT3/FT4 ratio was further assessed using correlation analysis.

**Result:**

A cohort of 251 patients with AIS was recruited; among them, 46 patients experienced HT following IVT. Multivariate regression analysis presented a notable correlation between the FT3/FT4 ratio and the occurrence of HT, with an OR of 0.444 [95% CI 0.209−0.946, *p* = 0.035]. The area under the ROC curve (AUC) of the FT3/FT4 ratio for discriminating HT from non‐HT was 0.774 (95% CI 0.695−0.853, *p* < 0.001), with an optimal cutoff value of 0.211. Correlation analysis demonstrated that FT3/FT4 levels were negatively related to baseline NIHSS score (*r* = −0.317, *p* = 0.032), In patients with HT, the NIHSS score at 24 h following thrombolysis (*r* = −0.367, *p* = 0.012) and the mRS score at 3 months post‐onset (*r* = −0.394, *p* = 0.007) demonstrated significant correlations.

**Conclusion:**

In patients with AIS, lower FT3/FT4 levels were found to be a potential predictive marker for HT. Nevertheless, no association was observed between FT3/FT4 levels and HT types. Moreover, lower FT3/FT4 levels were indicative of poorer clinical outcomes in HT patients.

## Graphical abstract

The research consecutively enrolled acute ischemic stroke (AIS) patients receiving intravenous thrombolysis (IVT) therapy. A cohort of 251 patients with AIS was recruited at. Clinical information, including free triiodothyronine to free thyroxine (FT3/FT4) from patients, was gathered, and data analysis was conducted. We found that the hemorrhagic transformation (HT) group demonstrated significantly reduced FT3/FT4 levels relative to non‐HT. Nevertheless, no association was observed between FT3/FT4 levels and HT types. Moreover, lower FT3/FT4 levels were indicative of poorer clinical outcomes in HT patients.

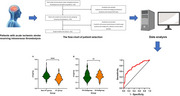

## 1 Introduction

Stroke ranks as a primary cause of mortality and disability globally (Zhou et al. 2019). In China, the stroke burden is particularly heavy. A recent national survey estimated that in 2020, there were approximately 17.8 million prevalent stroke cases, 3.4 million new incident cases, and 2.3 million stroke‐related deaths among adults aged 40 and older. Notably, acute ischemic stroke (AIS) constituted 86.8% of all incident strokes (Tu et al. 2023). Both vascular occlusion and cerebral hypoperfusion can result in AIS (Prabhakaran et al. 2015). Intravenous thrombolysis (IVT) is the standard treatment for AIS, leading to improved neurological outcomes. Nevertheless, some patients experience early neurological deterioration (Tisserand et al. 2014) and suffer from hemorrhagic transformation (HT) after IVT (Ma et al. 2021). Consequently, identifying biological markers to predict short‐term post‐IVT in‐hospital outcomes in AIS patients is essential for risk stratification for HT and prognostic assessment.

Thyroid hormones serve as neuro‐regulators during the ontogeny of the central nervous system (CNS) and are critical for neurocognitive function following CNS maturation (Calzà et al. 2015). A prospective clinical study found that a temporary decline in triiodothyronine concentrations after AIS could be associated with the extent of neurological damage (Sidorov et al. 2023). A meta‐analysis suggests that diminished triiodothyronine levels may predict a poor prognosis after AIS. Conversely, higher thyroxine levels correlate with worse outcomes (Jiang et al. 2017). These findings collectively indicate that thyroid hormone status is closely linked to stroke progression and outcome.

In recent years, the free triiodothyronine (FT3) to free thyroxine (FT4) ratio has garnered considerable attention as an indirect marker reflecting FT4 to FT3 conversion and peripheral deiodinase activity. This ratio is considered a more accurate indicator of thyroid function than traditional thyroid hormone level in serum, as it better represents the actual physiological state of thyroid function (Maia et al. 2011). Currently, it has been confirmed that FT3/FT4 are closely related to the conventional risk factors of AIS, including hypertension, diabetes (Mehran et al. 2022), and dyslipidemia (Sun et al. 2022). In recent clinical studies, FT3/FT4 may be found to be a potential prognostic biomarker indicating a worse outcome in non‐obstructive coronary myocardial infarction (Gao et al. 2021) and chronic kidney disease (Zhang et al. 2022) in glomerular crescent patients with normal thyroid function. In addition, recent research has reported that both FT3 level and FT3/FT4 ratio may be crucial indicators in predicting the occurrence of early neurological deterioration (END) in AIS patients (Rao et al. 2025). Notably, hemorrhagic transformation (HT) is one of the most critical and feared causes of END following intravenous thrombolysis. These findings collectively indicate that thyroid hormone status is closely linked to stroke progression and outcome. Nevertheless, the relation between the FT3/FT4 ratio and HT after intravenous thrombolysis is still not well understood in euthyroid individuals with AIS.

The objective of the research was to examine the relation between the FT3/FT4 ratio and HT following IVT in AIS individuals, potentially contributing additional clinical and scientific data for the management and prognostic evaluation of AIS patients receiving IVT.

## 2 Method

### 2.1 Study Population

The research was conducted as a single‐center, retrospective analysis. It included patients with AIS who received IVT at the Affiliated Hospital of Qingdao University between January 2020 and August 2024. They were included in the study. Inclusion criteria were (1) AIS diagnosis confirmed by experienced neurologists referring to the 2018 Chinese Guidelines for the Diagnosis and Treatment of AIS; (2) stroke onset within 4.5 h, and treatment with intravenous rt‐PA thrombolysis; and (3) participants were required to be ≥18 years old with written informed consent. The exclusion criteria included (1) patients with thyroid diseases or a history of thyroid surgery that could affect FT3 and FT4 levels; (2) patients with missing key data in their medical records, precluding complete analysis; (3) patients who underwent bridging endovascular therapy such as mechanical thrombectomy; and (4) patients with pre‐existing neurological deficits (mRS >2) from prior strokes. This study adhered to the Declaration of Helsinki, and ethical approval was from the ethics committee of the Affiliated Hospital of Qingdao University (Approval NO. QYFYWZLL29541).

### 2.2 Data Collection

Researchers retrospectively harvested data for each patient using a standardized form. Demographic characteristics included age and sex. Vascular factors comprised hyperlipidemia, abnormality in coronary artery, hypertension, atrial fibrillation, habitual intake of alcohol, diabetes, and smoking. Baseline clinical data included systolic blood pressure (SBP), diastolic blood pressure (DBP), pulse, and onset‐to‐treatment time (OTT). Evaluations comprised National Institutes of Health Stroke Scale (NIHSS) scores at baseline and post‐IVT 24 h and Modified Rankin Scale (mRS) scores at 3 months post‐discharge. Stroke subtypes were categorized based on Trial of Org 10172 in Acute Stroke Treatment (TOAST) criteria (Adams et al. 1993); Laboratory data comprised admission glucose, white blood cell count, platelets, INR, and fibrinogen when the patient arrived in the emergency room. Additionally, fasting blood tests on the second day of admission consisted of triglycerides (TG), uric acid, high‐density lipoprotein (HDL), homocysteine (HCY), thyroid function tests, low‐density lipoprotein (LDL), and total cholesterol (TC), which were also taken into consideration. Thyroid function tests included thyroid‐stimulating hormone receptor antibody (TRAb), FT3, thyroid peroxidase antibody (TPOAb), thyroglobulin, thyroid‐stimulating hormone (TSH), FT4, and anti‐thyroglobulin antibody (TgAb). The FT3 to FT4 ratio is denoted as FT3/FT4.

### 2.3 Intravenous Thrombolysis

Alteplase IVT was performed in accordance with international guidelines. A dose of 0.9 mg/kg body weight was used, capped at a maximum of 90 mg. The drug was administered via a two‐stage infusion protocol. First, 10% of the high dose was infused within 1 min, and then the remaining 90% dosage was infused within 60 min.

### 2.4 Clinical Outcome

The main endpoint was HT. HT was identified as bleeding found by follow‐up CT or MRI during 7 days post‐IVT, in line with the definition from the European Cooperative Acute Stroke Study (ECASS) II definition (Álvarez‐Sabín et al. 2013); According to CT imaging characteristics, HT was classified into parenchymal hemorrhage (PH) and hemorrhagic infarction (HI) (Hacke et al. 1995). HI was characterized by the presence of petechiae within the infarcted region, while PH was defined as space‐occupying hemorrhage, representing a more severe form of HT. Imaging interpretations were adjudicated by two experienced neurologists, with discrepancies resolved through consensus with a third neurologist. The secondary endpoint was early neurological deterioration (END) and long‐term prognosis at 3 months after discharge. END was characterized by an elevation of at least 4 points in the NIHSS score from admission or death within 24 h post‐IVT (Yu et al. 2020); the long‐term prognosis was assessed by the mRS score, with an mRS score of <3 indicating a favorable prognosis and an mRS score of ≥3 suggesting a poor prognosis (Ali et al. 2013).

### 2.5 Statistical Analysis

The Kolmogorov–Smirnov test was applied for evaluation on whether quantitative data distribution was normal. Normally distributed data were reported as mean ± standard deviation (SD), and group comparisons were accomplished by independent samples *t*‐tests. Non‐normally distributed data were presented as median (interquartile range, IQR), and intergroup comparisons were performed using the Mann–Whitney test. Categorical data were presented as percentages, and the Pearson chi‐square test was utilized. We first conducted a univariate analysis and then incorporated confounders with *p*‐value < 0.05 into a multivariate logistic analysis to build Model 1, and added vascular risk factors (hypertension, AF) and demographic factors (age, gender) into Model 2 for adjustment. The predictive performance of these factors was assessed using ROC analysis, with AUC computed to evaluate diagnostic accuracy. The Youden index was employed to assess the optimal cut‐off point to identify HT. Spearman correlation analyses were conducted to determine the relation between clinical outcomes and FT3/FT4 levels in HT individuals. Statistical analysis was accomplished via IBM SPSS 25.0 (IBM Corporation, Armonk, NY, USA). Due to the small magnitude of the original values of the FT3/FT4 level (ranging from 0.102 to 0.509 with a mean value of 0.253), we scaled the outcome variable by multiplying it by 100 to improve the interpretability of the logistic regression coefficients before performing logistic analyses. Its ratio and 95% CI are based on the amplified variables.

## 3 Results

### 3.1 Characteristics of Included Patients

**FIGURE 1:**
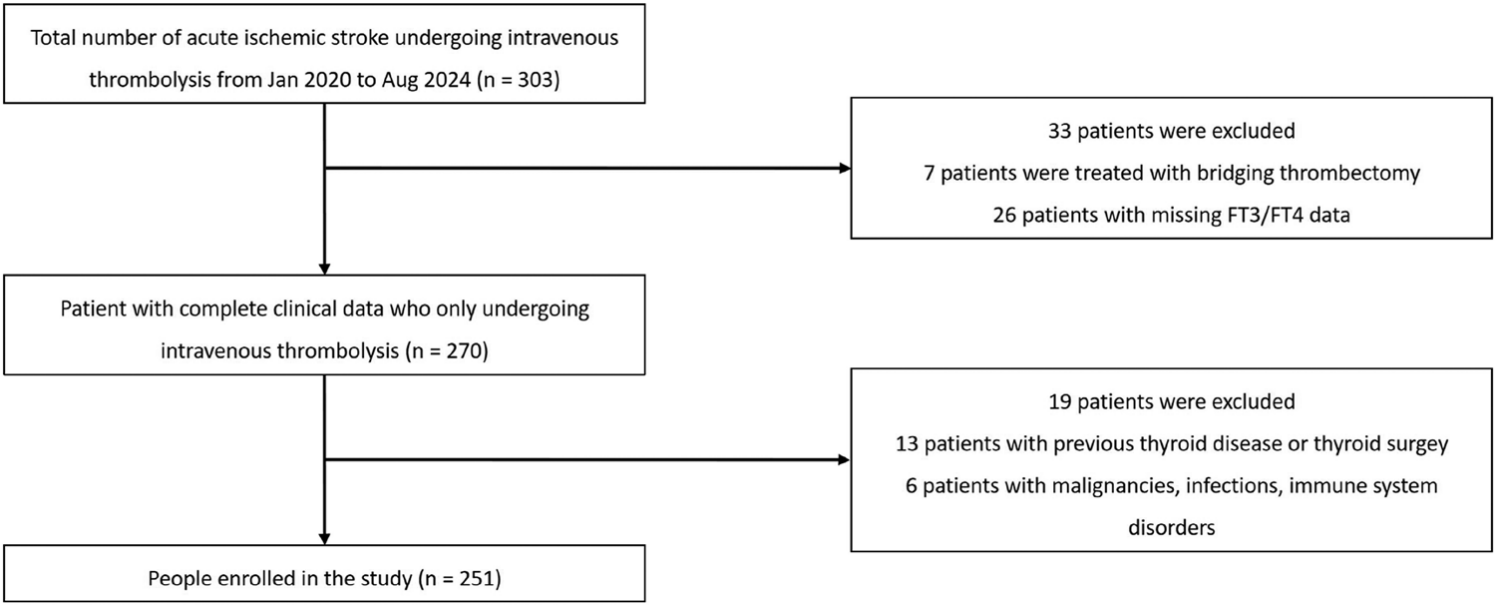
The flow chart of patient selection.

303 patients were initially included. Exclusions were made as follows: 7 patients due to bridging artery thrombolysis, 26 due to refusal to complete thyroid function tests resulting in missing data for FT3 and FT4, 13 due to prior thyroid disease or surgery, and 6 due to malignancies, infections, or immune system disorders. Ultimately, 251 patients were enrolled within the final step of analysis. Figure 1 outlines the subject recruitment. The mean age of enrolled patients was 65 ± 11 years, with 163 males (64.94%). Among the 46 patients who experienced HT, the average age was 66 ± 12 years, and 60.87% were male. Baseline data for the enrolled patients are presented in Table 1. We observed that OTT, NIHSS scores before IVT, pulse, admission glucose, WBC, and admission glucose levels were markedly elevated in the HT group relative to non‐HT cases. Conversely, FT3 and TC concentrations were substantially reduced in HT patients (all *p* < 0.05). Additionally, the TOAST classification system also revealed statistically significant intergroup variations (*p* < 0.05). We further stratified the population by FT3/FT4 ratio quartiles, as shown in Table 2. This analysis revealed several critical findings: a strong graded inverse association between the FT3/FT4 ratio and HT incidence (*p* < 0.001). Furthermore, patients in the lowest quartile exhibited a clinical profile with higher HT risk, including older age, higher NIHSS scores, higher pulse, and lower diastolic blood pressure compared to those in the highest quartile (all *p* < 0.05). Besides, the stroke subtypes also varied significantly (*p* < 0.05).

**TABLE 1:** Comparing baseline features in AIS patients with and without HT post‐IVT.

Variable	HT (*n* = 46)	Non‐HT (*n* = 205)	*p*‐value
Age (years), mean ± SD	66 ± 12	65 ± 11	0.496
Male, *n* (%)	28 (60.87)	143 (69.76)	0.376
OTT (min), median (IQR)	204 (171–245)	166 (112–194)	**<0.001**
SBP (mmHg), mean ± SD	142 ± 19	148 ± 22	0.075
DBP (mmHg), mean ± SD	83 ± 17	85 ± 17	0.519
Pulse (beats/min), mean ± SD	79 ± 13	71 ± 15	**0.001**
NIHSS scores before IVT, median (IQR)	9 (4–15)	4 (2–8)	**<0.001**
Stroke subtype, *n* (%)			**<0.001**
LAA	33 (71.74)	22 (10.73)	
SAO	3 (6.52)	95 (46.34)	
CE	10 (21.74)	124 (60.49)	
Hyperlipidemia, n (%)	14 (30.43)	58 (28.29)	0.772
Diabetes mellitus, n (%)	12 (26.09)	42 (20.49)	0.404
Hypertension, *n* (*n*%)	27 (58.70)	126 (61.46)	0.728
Coronary artery disease, *n* (%)	12 (26.09)	35 (17.07)	0.157
Atrial fibrillation, *n* (%)	27 (58.70)	33 (16.10)	0.110
Smoking, *n* (%)	16 (34.78)	95 (46.34)	0.154
Drinking, *n* (%)	11 (23.91)	77 (37.56)	0.080
Thyroglobulin (ng/mL), median (IQR)	10.42 (4.35–14.52)	10.25 (5.16–16.59)	0.456
FT3 (pmol/L), mean ± SD	3.16 ± 0.84	3.99 ± 0.73	**<0.001**
FT4 (pmol/L), mean ± SD	15.69 ± 3.09	15.37 ± 2.42	0.440
FT3/FT4, mean ± SD	0.206 ± 0.057	0.264 ± 0.056	**<0.001**
TSH (uIU/mL), median (IQR)	1.56 (0.92–2.41)	1.55 (0.94–2.36)	0.819
TgAb (U/mL), median (IQR)	13.07 (10.58–17.63)	13.00 (10.00–15.2)	0.318
TPOAb (U/mL), median (IQR)	13.1 (10.6–17.6)	9.72 (9.00–14.29)	0.470
TRAb (IU/L), median (IQR)	0.80 (0.40–0.83)	0.80 (0.30–0.82)	0.697
admission glucose (mmol/L), median (IQR)	7.68 (6.15–10.00)	6.71 (5.92–8.06)	**0.035**
WBC (*10^9^/L), median (IQR)	7.92 (6.88–9.20)	7.02 (5.86–8.70)	**0.031**
CRP, median (IQR)	0.50 (0.50–1.28)	0.50 (0.50–1.79)	0.448
Platelet (*10^9^/L), mean ± SD	207 ± 59	205 ± 50	0.838
INR, mean ± SD	1.07 ± 0.10	1.05 ± 0.11	0.261
Fibrinogen (g/l), mean ± SD	3.06 ± 0.65	2.96 ± 0.69	0.370
D‐dimer (ng/l), (IQR)	325 (228–540)	280 (195–420)	0.110
Fasting glucose (mmol/L), median (IQR)	5.68 (4.89–7.46)	5.11 (4.65–5.81)	**0.013**
TG (mmol/L), mean ± SD	1.08 ± 0.39	1.32 ± 0.65	**0.001**
TC (mmol/L), mean ± SD	4.53 ± 0.95	4.38 ± 1.12	0.398
HDL (mmol/L), mean ± SD	1.20 ± 0.27	1.24 ± 0.70	0.716
LDL (mmol/L), mean ± SD	2.77 ± 0.80	2.62 ± 0.85	0.251
Uric acid (µmmol/L), mean ± SD	270.69 ± 120.17	307.16 ± 75.52	0.054
Homocysteine (mmol/L), median (IQR)	12.3 (9.73–15.28)	11.7 (10.01–14.62)	0.825

**Abbreviations**: DBP, diastolic blood pressure; HDL, high density lipoprotein; HT, hemorrhagic transformation; IQR, interquartile range; LDL, low density lipoprotein; NIHSS, National Institutes of Health Stroke Scale; SBP, systolic blood pressure; SD, standard deviation; TC, total cholesterol; TG, triglyceride; TgAb, anti‐thyroglobulin antibody; TPOAb, thyroid peroxidase antibody; TRAb, thyroid‐stimulating hormone receptor antibody; TSH, thyroid‐stimulating hormone; WBC, white blood cells.

**TABLE 2:** Baseline characteristics of the study population grouped by FT3/FT4 ratio.

Variable	Q1 (*n* = 63) (FT3/FT4 < 0.214)	Q2 (*n* = 63) (0.214 ≤ FT3/FT4 < 0.250)	Q3 (n = 62) (0.250 ≤ FT3/FT4 < 0.291)	Q4 (*n* = 63) (FT3/FT4 ≥ 0.291)	*p*‐value
Age (years), mean ± SD	68 ± 11	69 ± 10	63 ± 11	61 ± 11	**<0.001**
Male, *n* (%)	38 (60.32)	41 (65.08)	46 (74.19)	47 (74.60)	0.229
OTT (min), median (IQR)	185 (137–237)	146 (120–191)	171 (114–206)	171 (108–202)	0.102
SBP (mmHg), mean ± SD	142 ± 20	148 ± 24	151 ± 23	149 ± 20	0.153
DBP (mmHg), median (IQR)	80 (69–89)	79 (71–91)	88 (74.8–98.0)	87 (78–98)	**0.004**
Pulse (beats/min), median (IQR)	75 (67–87)	70.0 (61–78)	70 (62–77)	70 (64–81)	**0.013**
NIHSS scores before IVT, median (IQR)	10 (4–16)	6 (2–10)	5 (2–7)	4 (2–6)	**<0.001**
Stroke subtype, *n* (%)					**<0.001**
LAA	36 (57.14)	31 (49.21)	29 (46.77)	28 (44.44)	
SAO	12 (19.05)	21 (33.33)	28 (45.16)	34 (53.97)	
CE	15 (23.81)	11 (17.46)	5 (8.06)	1 (1.59)	
HT	28 (44.44)	8 (12.70)	6 (9.68)	4 (6.35)	**<0.001**
Hyperlipidemia, *n* (%)	17 (26.98)	21 (33.33)	14 (22.58)	20 (31.75)	0.537
Diabetes mellitus, *n* (%)	14 (22.22)	10 (15.87)	13 (20.97)	17 (26.98)	0.506
Hypertension, *n* (*n*%)	39 (61.90)	36 (57.14)	46 (74.19)	32 (50.79)	0.052
Coronary artery disease, *n* (%)	8 (12.70)	16 (25.40)	11 (17.74)	12 (19.05)	0.335
Atrial fibrillation, *n* (%)	11 (17.46)	9 (14.29)	13 (20.97)	12 (19.05)	0.796
Smoking, *n* (%)	24 (38.10)	29 (46.03)	26 (41.94)	32 (50.79)	0.517
Drinking, *n* (%)	18 (28.57)	18 (28.57)	24 (38.71)	28 (44.44)	0.163
Thyroglobulin (ng/mL), median (IQR)	11.22 (4.70–14.5)	9.31 (5.20–16.5)	8.18 (4.10–13.8)	11.20 (5.80–20.1)	0.312
FT3 (pmol/L), mean ± SD	2.97 ± 0.63	3.77 ± 0.46	4.08 ± 0.61	4.53 ± 0.64	**<0.001**
FT4 (pmol/L), mean ± SD	16.61 ± 2.89	16.06 ± 1.82	15.19 ± 2.31	13.85 ± 2.21	**<0.001**
TSH (uIU/mL), median (IQR)	1.72 (1.30–2.50)	1.54 (0.90–2.30)	1.51 (0.90–2.50)	1.18 (0.80–2.10)	0.086
TgAb (U/mL), median (IQR)	12.48 (10.10–15.20)	13.50 (11.10–16.30)	13.01 (10.10–16.40)	12.70 (10.00–14.30)	0.265
TPOAb (U/mL), median (IQR)	9.49 (9.00–14.40)	10.20 (9.00–16.10)	10.70 (9.00–13.90)	9.00 (9.00–13.70)	0.417
TRAb (IU/L), median (IQR)	0.80 (0.40–0.80)	0.80 (0.30–0.80)	0.80 (0.30–0.90)	0.80 (0.40–0.80)	0.965
Admission glucose (mmol/L), median (IQR)	6.64 (6.00–8.50)	6.39 (5.90–7.50)	6.77 (6.00–8.10)	7.01 (6.00–8.70)	0.283
WBC (*109/L), median (IQR)	7.83 (6.50–9.30)	6.95 (5.80–8.40)	6.95 (5.90–8.40)	7.21 (5.90–9.20)	0.125
CRP, median (IQR)	0.50 (0.50–1.50)	0.50 (0.50–1.60)	0.50 (0.50–1.50)	0.60 (0.50–3.00)	0.602
Platelet (*109/L), median (IQR)	196 (169–237)	214 (168–243)	205 (176–247)	187 (162–219)	0.169
INR, median (IQR)	1.05 (1.00–1.10)	1.03 (1.00–1.10)	1.04 (1.00–1.1)	1.06 (1.00–1.10)	0.513
Fibrinogen (g/l), median (IQR)	2.86 (2.60–3.4)	2.84 (2.50–3.30)	2.88 (2.50–3.50)	2.91 (2.50–3.20)	0.886
D‐dimer (ng/l), median (IQR)	280 (200–420)	270 (210–420)	270 (160–413)	340 (200–460)	0.620
Fasting glucose (mmol/L), median (IQR)	5.20 (4.70–6.10)	5.08 (4.50–5.80)	5.12 (4.80–5.90)	5.21 (4.90–6.60)	0.633
TG (mmol/L), median (IQR)	1.12 (0.80–1.50)	1.18 (0.80–1.70)	1.10 (0.80–1.50)	1.11 (0.90–1.50)	0.817
TC (mmol/L), median (IQR)	4.40 (3.50–5.00)	4.43 (3.70–5.30)	4.47 (3.60–5.10)	4.35(3.60‐5.10)	0.850
HDL (mmol/L), median (IQR)	1.14 (1.00–1.30)	1.17 (1.00–1.30)	1.21 (1.10–1.40)	1.20 (1.00–1.30)	0.427
LDL (mmol/L), median (IQR)	2.60 (1.90–3.00)	2.77 (2.00–3.30)	2.62 (2.00–3.10)	2.60 (2.00–3.30)	0.811
Uric acid (µmmol/L), median (IQR)	290.80 (226.30–347.70)	296.40 (243.20–358.40)	302.10 (242.40–365.30)	305.60 (253.20–341.20)	0.656
Homocysteine (mmol/L), median (IQR)	11.90 (10.20–15.00)	11.65 (10.00–14.50)	11.80 (10.20–14.20)	12.20 (9.70–16.00)	0.937

**Abbreviations**: DBP, diastolic blood pressure; HDL, high density lipoprotein; HT, hemorrhagic transformation; IQR, interquartile range; LDL, low density lipoprotein; NIHSS, National Institutes of Health Stroke Scale; SBP, systolic blood pressure; SD, standard deviation; TC, total cholesterol; TG, triglyceride; TgAb, anti‐thyroglobulin antibody; TPOAb, thyroid peroxidase antibody; TRAb, thyroid‐stimulating hormone receptor antibody; TSH, thyroid‐stimulating hormone; WBC, white blood cells.

### 3.2 Relationship Between FT3/FT4 and HT After IVT

**FIGURE 2:**
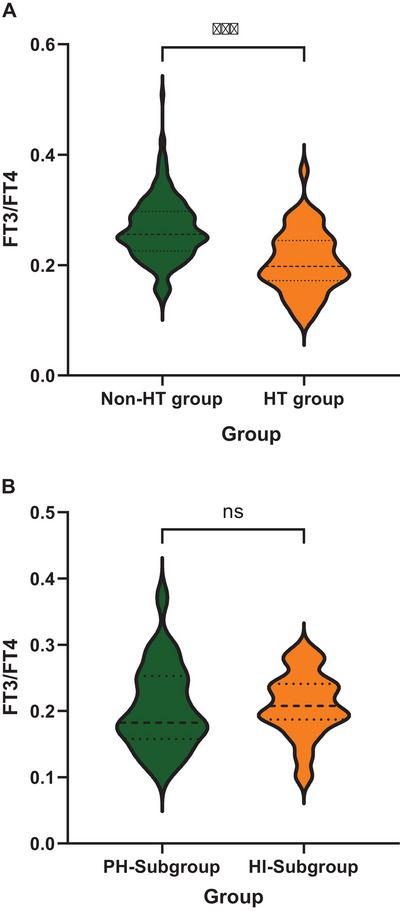
Violin plot comparison of FT3/FT4 levels in different diagnostic groups. **(A)** Comparison of FT3/FT4 level in HT and Non‐HT groups and **(B)** Comparison of plasma Sema3A level in PH and HI subgroups in the HI group. **Abbreviations**: HI, hemorrhagic infarction; HT, hemorrhagic transformation; PH, parenchymal hemorrhage. ^***^ *p* < 0.001, ^**^ *p* < 0.01, and ^*^ *p* < 0.05.

The HT group demonstrated significantly reduced FT3/FT4 levels relative to non‐HT (0.264 ± 0.056 vs. 0.205 ± 0.057, *p* < 0.001; see Figure 2A and Table 1). As illustrated in Table 3, initial screening through univariate logistic regression revealed several significant variables (*p* < 0.05), which were then incorporated into a multivariate regression analysis for comprehensive adjustment. After incorporating the above variables with *p* < 0.05 into a multivariate logistic regression model and adjusting for demographic characteristics and vascular risk factors, the results revealed that the FT3/FT4 remained an independent protective factor for HT (OR = 0.826; 95% CI: 0.746‐0.914; *p* < 0.001).

**TABLE 3:** Exploring predictors of HT post‐IVT within AIS individuals using univariate and multivariate analyses.

Variables	Univariate analysis		Multivariate analysis (model 1)		Multivariate analysis (model 2)	
	OR (95%CI)	*p*‐value	OR (95%CI)	*p*‐value	OR (95%CI)	*p*‐value
Age	1.010 (0.982–1.039)	0.495			0.811 (0.310–2.505)	0.812
Male	1.352 (0.693–2.639)	0.377			1.011 (0.969–1.054)	0.619
OTT	1.015 (1.008–1.002)	**<0.001**	1.012 (1.012–1.020)	**0.004**	1.103 (1.004–1.021)	**0.005**
SBP	0.986 (0.972–1.001)	0.077				
DBP	0.993 (0.973–1.014)	0.518				
Pulse	1.673 (1.186–2.359)	**0.003**	1.040 (1.007–1.073)	**0.016**	1.044 (1.010–1.080)	**0.012**
NIHSS scores before IVT	1.858 (1.374–2.513)	**<0.001**	1.035 (0.964–1.111)	0.348	1.028 (0.954–1.107)	0.473
Stroke subtype						
LAA	Reference		Reference			Reference
SAO	0.798 (0.342–1.861)	0.601	1.505 (0.464–4.879)	0.496	1.635 (0.485–5.506)	0.428
CE	0.072 (0.018–0.283)	**<0.001**	0.165 (0.032–0.865)	**0.033**	0.159 (0.029–0.879)	**0.035**
Hyperlipidemia	0.902 (0.449–1.812)	0.772				
Diabetes	0.730 (0.348–1.531)	0.405				
Hypertension	0.891 (0.465–1.708)	0.728			2.676 (1.001–7.151)	0.050
Atrial fibrillation	0.544 (0.255–1.158)	0.114			0.615 (0.202–1.869)	0.391
Coronary artery disease	0.583 (0.275–1.237)	0.160				
Smoking	1.619 (0.832–3.152)	0.156				
Drinking	1.914 (0.919–3.988)	0.083				
Thyroglobulin	0.980 (0.950–1.010)	0.186				
* FT3/FT4	0.285 (0.182–0.446)	**<0.001**	0.837 (0.761–0.921)	**<0.001**	0.826 (0.746–0.914)	**<** **0.001**
TSH	0.981 (0.898–1.073)	0.676				
TgAb	1.014 (0.991–1.038)	0.236				
TPOAb	0.994 (0.975–1.013)	0.519				
TRAb	0.883 (0.505–1.542)	0.661				
Admission glucose	1.096 (1.008–1.191)	**0.032**	1.058 (0.865–1.295)	0.581	1.054 (0.868–1.280)	0.596
WBC	1.037 (0.932–1.153)	0.507				
CRP	0.992 (0.961–1.025)	0.640				
Platelet	1.001 (0.995–1.007)	0.818				
INR	4.328 (0.320–58.547)	0.270				
Fibrinogen	1.229 (0.783–1.929)	0.369				
D‐dimer	1.0001 (0.9998–1.0005)	0.486				
Glucose	1.219 (1.054–1.409)	**0.008**	1.317 (0.980–1.770)	0.068	1.414 (1.032–1.939)	**0.031**
TG	0.406 (0.194–0.849)	**0.017**	0.276 (0.102–0.747)	**0.011**	0.267 (0.093–0.769)	**0.014**
TC	1.132 (0.850–1.509)	0.397				
HDL	0.878 (0.427–1.805)	0.723				
LDL	1.242 (0.858–1.797)	0.251				
Uric acid	0.994 (0.990–0.999)	0.010	0.997 (0.992–1.001)	0.154	0.997 (0.992–1.002)	0.221
Homocysteine	1.006 (0.983–1.030)	0.608				

*The OR and 95% CI were based on the amplified outcome variable (original value × 100).

Model 1 Adjusted for those with a *p* < 0.05 in the univariable regression.

Model 2 Adjusted for variables in model 1 plus age, sex, hypertension, and atrial fibrillation.

**Abbreviations**: DBP, diastolic blood pressure, HDL, high density lipoprotein, HT, hemorrhagic transformation, IQR, interquartile range, LDL, low density lipoprotein, NIHSS, National Institutes of Health Stroke Scale, SBP, systolic blood pressure, SD, standard deviation; TC, total cholesterol; TG, triglyceride; TgAb, anti‐thyroglobulin antibody; TPOAb, thyroid peroxidase antibody; TRAb, thyroid‐stimulating hormone receptor antibody; TSH, thyroid‐stimulating hormone; WBC, white blood cells.

### 3.3 Association Between FT3/FT4 and HT Subtypes

We further stratified HT into HI and PH groups and found that the PH patients exhibited significantly elevated thyroglobulin level (*p* = 0.022, Table 4), D‐dimer level (*p* = 0.001), and CRP level (*p* = 0.024), along with a higher NHISS score (*p* = 0.020). No statistically significant difference was observed in the FT3/FT4 ratio when comparing two cohorts (*p* = 0.780) (Figure 2B).

**TABLE 4:** Baseline demographic and clinical features categorized by HT types.

Variable	PH (*n* = 27)	HI (*n* = 19)	*p* ‐value
Age (years), median (IQR)	65 (58–71)	64 (61–79)	0.447
Male, *n* (%)	17 (65.38)	13 (68.42)	0.989
OTT (min), mean ± SD	197 ± 44	220 ± 54	0.470
SBP (mmHg), mean ± SD	139 ± 17	145 ± 21	0.186
DBP (mmHg), median (IQR)	78 (68–90)	82 (78–92)	0.294
Pulse (beats/min), mean ± SD	78 ± 11	83 ± 16	0.811
NIHSS scores before IVT, median (IQR)	10 (6–16)	5 (2–12)	**0.020**
Stroke subtype, *n* (%)			0.657
LAA	18 (66.67)	15 (78.95)	
SAO	2 (7.41)	1 (5.26)	
CE	7 (25.93)	3 (15.79)	
Hyperlipidemia, *n* (%)	7 (26.92)	7 (36.84)	0.428
Diabetes mellitus, *n* (%)	6 (23.08)	6 (31.58)	0.477
Hypertension, *n* (*n*%)	17 (65.38)	10 (52.63)	0.483
Atrial fibrillation, *n* (%)	7 (26.92)	5 (26.32)	0.618
Coronary artery disease, *n* (%)	6 (22.22)	6 (31.58)	0.477
Smoking, *n* (%)	8 (30.77)	8 (42.11)	0.382
Drinking, *n* (%)	5 (19.23)	6 (31.58)	0.307
Thyroglobulin (ng/mL), median (IQR)	8.03 (3.17–14.05)	13.1 (6.51–19.91)	**0.022**
FT3 (pmol/L), mean ± SD	3.07 ± 0.91	3.31 ± 0.73	0.360
FT4 (pmol/L), mean ± SD	15.30 ± 2.45	15.92 ± 3.82	0.306
FT3/FT4, mean ± SD	0.204 ± 0.063	0.208 ± 0.049	0.780
TSH (uIU/mL), median (IQR)	1.55 (0.96–2.69)	1.68 (0.80–2.40)	0.894
TgAb (U/mL), median (IQR)	13.47 (10.10–18.00)	12.50 (10.74–14.80)	0.728
TPOAb (U/mL), median (IQR)	10.08 (9.00–14.20)	11.50 (9.00–14.10)	0.945
TRAb (IU/L), median (IQR)	0.80 (0.44–0.92)	0.80 (0.30–0.80)	0.593
Admission glucose (mmol/L), median (IQR)	7.60 (6.07–8.65)	8.08 (6.18–11.77)	0.806
WBC (*10^9^/L), mean ± SD	8.01 ± 1.90	8.07 ± 2.02	0.762
CRP, median (IQR)	0.87 (0.50–3.62)	0.50 (0.50–0.50)	**0.024**
Platelet (*10^9^/L), mean ± SD	203 ± 56	200 ± 61	0.564
INR, mean ± SD	1.06 ± 0.09	1.09 ± 0.11	0.370
Fibrinogen (g/l), median (IQR)	3.11 (2.69–3.80)	2.79 (2.64–3.06)	0.124
D‐dimer (ng/l), median (IQR)	340 (180‐620)	310 (240‐440)	**0.001**
Fasting glucose (mmol/L), median (IQR)	5.47 (4.79‐6.88)	6.14 (4.92‐7.53)	0.224
TG (mmol/L), median (IQR)	1.04 (0.77‐1.41)	0.94 (0.81‐1.40)	0.956
TC (mmol/L), mean ± SD	4.37 ± 0.91	5.00 ± 0.99	0.184
HDL (mmol/L), median (IQR)	1.22 (1.02‐1.39)	1.13 (0.95‐1.37)	0.616
LDL (mmol/L), mean ± SD	2.59 ± 0.71	3.03 ± 0.88	0.073
Uric acid (µmmol/L), median (IQR)	245.00 (182.0‐365.30)	244.30 (190.70‐294.00)	0.956
Homocysteine (mmol/L), median (IQR)	12.49 (9.30‐15.75)	11.9 (9.80‐16.10)	0.858

**Abbreviations**: DBP, diastolic blood pressure; HDL, high density lipoprotein; HT, hemorrhagic transformation; IQR, interquartile range; LDL, low density lipoprotein; NIHSS, National Institutes of Health Stroke Scale; SBP, systolic blood pressure; SD, standard deviation; TC, total cholesterol; TG, triglyceride; TgAb, anti‐thyroglobulin antibody, TPOAb, thyroid peroxidase antibody; TRAb, thyroid‐stimulating hormone receptor antibody; TSH. thyroid‐stimulating hormone; WBC, white blood cells.

### 3.4 FT3/FT4 Ratio Predictive Value for HT After IVT

**FIGURE 3:**
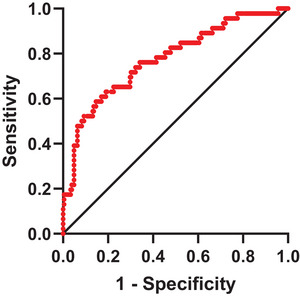
ROC analysis of FT3/FT4 for predicting HT after IVT.

To evaluate the prognostic capability of the FT3/FT4 ratio for post‐IVT, we performed ROC analysis (Figure 3). The AUC for distinguishing HT from non‐HT was 0.774 (*p* < 0.001, 95% CI: 0.695–0.853), with an optimal cutoff value of 0.211 (sensitivity 85.4%, specificity 58.7%, maximum Youden index 0.441).

### 3.5 Relation Between FT3/FT4 and the Functional Outcomes

We first conducted a correlation analysis of FT3/FT4 with the outcomes in patients with HT. NIHSS score sand mRS scores were incorporated into the correlation analysis with FT3/FT4. The study revealed exhibited negative correlations with baseline NIHSS (*r* = −0.317, *p* = 0.032, Table 5), NIHSS score after IVT (*r* = −0.367, *p* = 0.012, Table 5), and mRS score after 3 months post‐discharge (*r* = −0.394, *p* = 0.007, Table 5).

**TABLE 5:** Correlation of FT3/FT4 level with clinical characteristics of HT groups.

Variable	*r*	*p‐*value
Baseline NIHSS	−0.317	**0.032**
NIHSS score after IVT	−0.367	**0.012**
mRS score after 3 months	−0.394	**0.007**

Additionally, we explored relation between FT3/FT4 and both in‐hospital and 3‐month outcomes across the entire cohort. Of 251 subjects enrolled within this research, 22 patients (8.76%) developed END within 24 h after IVT and 68 (27.09%) had a poor prognosis (mRS score ≥3) at 3 months post‐onset. After univariate logistic regression analysis and multifactorial adjustment, we found that FT3/FT4 level were significantly and negatively related to END risk (OR 0.850, 95% CI 0.776–0.931, *p* < 0.001, Table 6). Similarly, the decrease FT3/FT4 level was significantly related to a favorable prognosis at 3 months (OR 0.870, 95% CI 0.802–0.903, *p* = 0.001, Table 6).

**TABLE 6:** Correlation between FT3/FT4 and secondary endpoint events.

Variables	Univariate analysis OR (95%CI) *p*‐value		Multivariate analysis OR (95%CI) *p*‐value	
Secondary endpoint 1				
*END vs. Non‐END	0.852 (0.781–0.929)	**<0.001**	0.850 (0.776–0.931)	**< 0.001**
Secondary endpoint 2				
*Good prognosis vs. Poor prognosis	0.803 (0.750–0.861)	**<0.001**	0.870 (0.802–0.903)	**0.001**

*The OR and 95% CI was based on the amplified outcome variable (original value × 100).

## 4 Discussion

This research explored the relation between FT3/FT4 levels and HT following IVT in AIS patients. Key findings include: In AIS patients undergoing IVT, a lower FT3/FT4 ratio was linked to HT occurrence. Multivariate logistic regression indicated that the FT3/FT4 ratio serves as an independent protective factor for HT. ROC curve analysis also showed predictive capacity of FT3/FT4 ratios for HT, with 0.211 as the optimal cut‐off value to differentiate HT from non‐HT cases. However, FT3/FT4 ratios did not significantly differ across various subtypes of HT. In addition, lower FT3/FT4 levels were related to higher pre‐ and post‐IVT NHISS scores and 3‐month mRS scores in HT patients. In all patients with AIS, FT3/FT4 levels were negatively related to the END and prognosis at 3 months post‐onset.

In this study, patients with HT exhibited significantly lower FT3/FT4 levels than those without HT. This finding is consistent with prior studies suggesting that lower FT3/FT4 or FT3 levels in AIS patients undergoing IVT may predict adverse early outcomes, including sICH (Qiu et al. 2017, Liu et al. 2016). In parallel, a clinical study involving 416 patients demonstrated that reduced thyroid hormone concentrations may predict an elevated risk of HT in individuals experiencing AIS (Huang et al. 2019). However, existing studies primarily focused on a single thyroid function indicator. Our study may more accurately reflect the relation between actual thyroid function status and HT risk in individuals treated with IVT rather than relying on a single FT3 level. FT3/FT4 better captures the balance between hormone precursor (FT4) and active hormone (FT3) and is basically not affected by plasma binding proteins. It can relatively accurately represent mild metabolic changes in thyroid hormones and may more accurately reflect tissue‐level thyroid hormone activity and conversion efficiency (Maia et al. 2011). Besides, the FT3/FT4 ratio reflects FT4 to FT3 conversion and peripheral deiodinase activity and may more accurately reflect tissue‐level thyroid hormone activity and conversion efficiency (Strich et al. 2016). Previous studies have suggested that the FT3/FT4 ratio is a superior prognostic marker in various clinical settings (acute myocardial infarction, stroke, dyslipidemia, etc.) compared to isolated hormone levels, as it integrates both production and peripheral metabolism of thyroid hormones (Huang et al. 2019, Kang et al. 2018, Lai et al. 2021).

Clinical evidence consistently demonstrates that hypothalamic‐pituitary‐thyroid (HPT) axis dysregulation commonly accompanies severe CNS diseases in critically ill populations. The most common pattern of HPT axis impairment is the reduction of serum FT3 concentration under normal thyroid function (Rodriguez‐Perez et al. 2008). This phenomenon principally stems from disrupted central regulation of the HPT axis, particularly diminished TRH secretion; altered peripheral metabolism of thyroid hormones; and impaired hormone‐receptor binding and transport mechanisms (Warner and Beckett 2010). In addition, the neural plasticity in the infarct core and the ischemic penumbra increases, and the neuroendocrine microenvironment is activated after stroke (Schwarz et al. 2003). Based on this, we speculate that during IVT, the dysfunction of the CNS and the difficulty in maintaining structural integrity of the infarct core and ischemic penumbra lead to a disruption of the balance in the neuroendocrine microenvironment, which in turn results in a decrease in FT3/FT4 level. However, the associations observed in our study are correlational and do not establish causality. An alternative explanation is that a lower FT3/FT4 ratio primarily serves as a marker of more severe stroke insult and greater systemic physiological stress, which itself is a well‐established risk factor for HT (Andrade et al. 2020). In this scenario, thyroid hormone alterations would be an epiphenomenon rather than a direct contributor to HT. Notwithstanding the explanation above, and given the well‐recognized correlation between post‐IVT HT and blood‐brain barrier (BBB) damage and alteplase‐induced reperfusion injury (Jickling et al. 2014), it is plausible that thyroid hormone status may play a moderating role. Furthermore, higher FT3/FT4 values represent higher sensitivity of peripheral tissues to thyroid hormones (Nie et al. 2020). A clinical study demonstrated that thyroid hormone deficiency may lead to vascular endothelial dysfunction, which in turn causes vascular damage and increased permeability (Taddei et al. 2003). Besides, previous observations suggest that thyroid hormone deficiency may be associated with cerebral edema or compression of the ventricular system in severe neurological injury (Ambrosius et al. 2011). The experimental studies have shown that thyroid hormones play a role in maintaining cerebrovascular endothelial function and tight junction integrity of the BBB. Reduced thyroid hormone signaling has also been linked to increased vascular permeability and inflammatory activation in ischemic brain injury models (Ullrich et al. 2025). Therefore, based on this previous literature, we hypothesize a potential pathway whereby a low FT3/FT4 ratio might exacerbate BBB disruption or impair endothelial repair following IVT. However, this mechanistic pathway is speculative and based on prior evidence, not on direct findings from our present clinical dataset. Future mechanistic studies incorporating advanced neuroimaging, such as permeability assessments or experimental models, are essential to validate these potential pathways and distinguish correlation from causation.

Based on the analysis above, we further investigated whether FT3/FT4 can differentiate between HT and non‐HT. The ROC analysis revealed an AUC of 0.774, with an optimal cutoff value of 0.211 for distinguishing between HT and non‐HT. Consequently, we posit that low levels of FT3/FT4 possess a specific predictive value for HT in AIS patients undergoing IVT, indicating the need for careful selection and administration of antiplatelet drugs following IVT. However, further analysis of HT subtypes revealed that there is no relationship between FT3/FT4 and HT types. We hypothesize that the observed fluctuations in thyroid hormone level may reflect the physiological stress response after an AIS (Lee and Farwell 2016), rather than a direct association with the extent of the cerebral hemorrhage. Furthermore, HT primarily results from localized brain injury, and its severity mainly depends on the location of the hemorrhage and the mass effects of the hematoma (Fesharaki‐Zadeh and Datta 2024), The physical damage stemming from the hemorrhage per se may extend beyond the regulatory scope of thyroid hormones.

In addition, our correlation analysis indicated a profound inverse correlation between FT3/FT4 level and NHISS score before and after IVT and the mRS score 3 months after discharge in HT patients. Bunevicius et al. also found that lower thyroid hormone level were closely related to a more severe baseline NHISS score, a more complex clinical course, and a higher mortality rate (2015). Lower FT3/FT4 ratios may aggravate neurological deficits by impairing vascular endothelial cell function and decreasing cerebral blood flow (Nomoto et al. 2019, Smith and Ain 1995). Moreover, a reduced FT3/FT4 ratio may persist in influencing the patient's neurological recovery due to the attenuated body's capacity to repair post‐thrombolytic injuries, thereby prolonging neurological recovery or potentially leading to further deterioration (Talhada et al. 2019). This indicates that closer attention should be given to the dynamic monitoring and evaluation of neurological regression in HT patients with lower FT3/FT4 levels.

Previous studies have concluded that reduced FT3 and FT3/FT4 levels can predict the poor early and 3‐month prognosis of patients with AIS (Alevizaki et al. 2007, Wang et al. 2017), This is similar to our analysis, which showed that FT3/FT4 levels were significantly and negatively associated with END risk and prognosis at 3 months of discharge in all patients. We hypothesize that the underlying mechanism may be attributed to the suppression of neurotrophic factor production by low FT3 levels, which hinders axonal growth and myelination, thereby impeding the recovery of nerve function and potentially exacerbating its deterioration (Zhang et al. 2022, Vancamp et al. 2020). These findings suggest that administering low‐dose thyroid hormone supplementation may be a viable strategy to increase FT3/FT4 levels, potentially improving neurological outcomes following thrombolysis.

Our study has several limitations that should be considered. Firstly, our research is a single‐center, retrospective observational study that is limited by a small sample size and a patient population with restricted diversity. Secondly, the use of admission‐based thyroid function level in this investigation overlooks the assessment of thyroid function at additional time points. Consequently, the study did not evaluate the effects of IVT on FT3/FT4 level nor monitor post‐discharge variations in FT3/FT4 level. For subsequent investigations, we advocate for longitudinal assessment of FT3 and FT4 concentrations to enhance the predictive validity of our findings. Moreover, FT3/FT4 levels could be affected by various factors such as genetic susceptibility, prior medical history, and drugs. Although we instituted strategies to mitigate these confounding variables, their complete elimination proved unfeasible. The findings warrant further validation through a multicenter prospective study involving a larger sample size and monitoring FT3 and FT4 levels at multiple time points. This would help clarify the causal relationship between FT3/FT4 and HT after IVT in AIS patients, as well as explore its potential therapeutic significance. Certain relevant clinical variables, such as cerebral small vessel disease (CSVD), which has been identified as a risk factor for hemorrhagic transformation in recent studies (Fang et al. 2025), were not assessed in our dataset. The lack of neuroimaging‐based CSVD evaluation may have introduced residual confounding and should be addressed in future prospective studies. To confirm these results, larger‐scale, multicenter, prospective longitudinal studies are needed, along with testing of FT3/FT4 at multiple time points, to further explain the mechanism of HT.

## 5 Conclusion

Our findings indicate that a lower FT3/FT4 level is linked to a higher risk of HT following IVT in AIS patients. FT3/FT4 may serve as an independent protective factor against HT, and a reduced FT3/FT4 level appears to have predictive value for HT after IVT. However, FT3/FT4 levels were not correlated with HT subtypes after IVT. Further validation of our conclusions necessitates large‐scale, multicenter, longitudinal investigations.

## Author Contributions

**Haoran Ma**: conceptualization, methodology, software; data curation, formal analysis, validation, investigation, visualization, writing – original draft, and resources. **Hong Li**: conceptualization, methodology, investigation, formal analysis, supervision, resources, writing – review and editing, visualization, and project administration. **Xinyi Fu**: conceptualization, methodology, software, data curation, investigation, validation; writing – original draft, and formal analysis. **Chenchen Wei**: writing – review and editing, resources, project administration, funding acquisition, visualization, supervision, validation, investigation, formal analysis, conceptualization and Methodology. **Aijun Ma**: investigation, validation, formal analysis, supervision, resources, project administration, writing – review and editing, visualization, funding acquisition, conceptualization, and methodology.

## Funding

This research was supported by the National Natural Science Foundation of China (81971111, 82371331, 82401545) and the Natural Science Foundation of Shandong Province (ZR2022QH363).

## Ethics Statement

This research was approved by the Ethics Committee of the Affiliated Hospital of Qingdao University, approval number (QYFYWZLL29541).

## Conflicts of Interest

The authors declare no conflicts of interest.

## Data Availability

All data generated or analyzed during this study are included in this article. Further inquiries can be directed to either corresponding author.
